# Prevalence of Self-Reported Pelvic-Floor-Related Symptoms Among Female Patients with Multiple Sclerosis

**DOI:** 10.3390/healthcare14193324

**Published:** 2026-10-06

**Authors:** Waad Alqurashi, Danah Alyahya

**Affiliations:** Department of Physical Therapy and Rehabilitation Sciences, Applied Medical Sciences College, Majmaah University, Majmaah 11952, Saudi Arabia; 441204338@s.mu.edu.sa

**Keywords:** multiple sclerosis, pelvic floor dysfunction, urinary incontinence, quality of life, Saudi Arabia

## Abstract

**Highlights:**

**What are the main findings?**

- A substantial burden of self-reported pelvic floor symptoms was observed among women with Multiple Sclerosis.
- Pelvic floor symptoms were associated with poorer physical and emotional quality of life.

**What are the implications of the main findings?**

- Routine pelvic floor screening should be integrated into multiple sclerosis care.
- Early multidisciplinary rehabilitation may improve patient outcomes and quality of life.

**Abstract:**

**Introduction**: Multiple sclerosis (MS) is more prevalent among females worldwide. The Expanded Disability Status Scale (EDSS) assesses MS progression, including disability related to pelvic floor dysfunction (PFD), such as bowel and bladder dysfunction. However, data on PFD symptoms in patients with MS remain limited. **Objective**: To evaluate the prevalence and impact of self- reported pelvic-floor-related symptoms among women with MS in Saudi Arabia and their effects on quality of life (QOL). **Methods**: A cross-sectional study was conducted among 112 female patients with MS (EDSS ≤ 7, age ≥ 18 years). The participants completed two validated self-reported questionnaires, the Pelvic Floor Distress Inventory (PFDI-20) and the Pelvic Floor Impact Questionnaire (PFIQ-7), to assess self-reported pelvic-floor-related symptoms and their impact on QOL in women with MS. **Results**: The most commonly reported symptoms were frequent urination (83%), urge incontinence (70.5%), and constipation (76.8%). Following adjustment for multiple comparisons, increasing age was positively associated with greater urinary symptom distress, overall pelvic-floor-related symptom burden, and greater urinary and prolapse related impact on QOL. In the multivariable analysis, age remained significantly associated with total PFDI-20 scores after adjustment for BMI, MS duration, and marital status. BMI, MS diagnosis duration, and marital status were not significantly associated with total PFDI-20 or PFIQ-7 scores in the multivariable models. **Conclusions**: Women with MS reported a substantial burden of pelvic-floor-related symptoms, particularly urinary and colorectal anal symptoms, which were associated with adverse effects on physical activities and emotional well-being. These findings highlight the importance of recognizing and assessing pelvic-floor-related symptoms as part of comprehensive care for women with MS.

## 1. Introduction

Multiple sclerosis (MS) is a complex and poorly understood neurological disorder characterized by progressive inflammation and demyelination within the central nervous system (CNS). It is characterized by episodic neurologic dysfunction, leading to various motor, sensory, and cognitive impairments that may negatively affect quality of life (QOL) [1]. MS is a major cause of neurological disability worldwide, affecting approximately 2.8 million people and is more common among females than males, with a female-to-male ratio as high as 4:1 worldwide [2]. It is commonly diagnosed during women’s childbearing years [3]. A recent study reported that the overall prevalence of MS in Saudi Arabia was 40.40 per100,000 population, with females accounting for 66.5% of cases and having a median age of 32 years [4].

MS can be monosymptomatic or polysymptomatic, depending on the location and nature of the lesion [5]. It can present with various clinical symptoms resulting from the involvement of sensory, motor, visual, and brainstem pathways, including muscle weakness, fatigue, spasticity, gait instability, ataxia, numbness or altered sensation, and bowel, bladder, and sexual dysfunction [5,6]. Numerous instruments are used to assess the severity and progression of MS. The expanded Disability Status Scale (EDSS) is one of the most widely used instruments and is considered the gold standard measure for summarizing and quantifying disability in patients with MS. The EDSS provides a standardized assessment of disability across different stages of the disease, and mainly focuses on the functional system [7]. The scoring system ranges from 0, indicating a normal neurological examination, to 10, indicating death due to MS [8].

Pelvic floor dysfunction (PFD), a functional system of EDSS, is characterized by a wide range of clinical symptoms, including urinary incontinence (UI), overactive bladder (OAB), fecal incontinence (FI), pelvic organ prolapse (POP), vaginal wind, sexual dysfunction, and several chronic pain syndromes [9]. The EDSS score for PFD ranges from 0 (normal) to 6 (loss of bladder and bowel functions); higher scores indicate more severe dysfunction [8]. The prevalence of PFD is influenced by numerous factors such as increasing age, obesity, smoking, mode of delivery, and parity [10]. In 2018, a study conducted in 13 different regions of Saudi Arabia to assess the prevalence of PFD among females attending primary healthcare centers reported that 36.3% of the subjects had a subtype of UI, 54.1% had OAB, and 23.4% had POP [11], demonstrating the burden of pelvic-floor-related symptoms among Saudi women.

Despite the known association between MS and autonomic dysfunction, to date, no published studies to date have specifically investigated the prevalence and impact of PFD on QOL among Saudi women with MS. This study aimed to address this gap by assessing the prevalence and burden of self-reported pelvic-floor-related symptoms and their impact on QOL using validated questionnaires. We hypothesized that pelvic-floor-related symptoms, particularly OAB, would be commonly reported among women with MS and would significantly affect their QOL.

## 2. Materials and Methods

### 2.1. Study Design and Participants

A cross-sectional, survey-based study was conducted among 112 Saudi women diagnosed with MS. Participants were recruited through four MS societies across different regions of Saudi Arabia, which collectively included approximately 3000 women. The study invitation was randomly distributed by the MS societies to approximately 370 potentially eligible participants. To ensure privacy and voluntary participation, data were collected using an online self-administered questionnaire. A total of 114 participants responded to and completed the questionnaire, yielding a response rate of approximately 30.8%. Participants’ medical histories were reviewed by the primary investigator to determine eligibility based on the predefined inclusion and exclusion criteria, including the EDSS eligibility criterion. Two participants were excluded due to pre-existing bladder conditions unrelated to MS, resulting in a final analytic sample of 112 participants. The principal investigator collected and managed all data to maintain confidentiality.

Ethical approval was obtained from the Majmaah University Ethics Committee (Approval No.: MUREC-Mar.10/COM-2024/8-6). Written informed consent was obtained from all participants prior to enrollment in the study.

An a priori sample size calculation was performed using the best available epidemiological estimates relevant to the target population. Because no published study was identified that reported the prevalence of UI or pelvic-floor-related symptoms specifically among Saudi women with MS, two Saudi population estimates were used to inform the calculation: the prevalence of UI among Saudi women (36.3%) [11] and the proportion of women among individuals with MS in Saudi Arabia (66.5%) [4]. These estimates were used as planning assumptions to approximate the target population of Saudi women with MS. Using the Fleiss, Levin, and Paik approach for comparing two proportions, with a 95% confidence level (α = 0.05) and 90% statistical power (β = 0.10), the calculated minimum total sample size was 112 participants using Epi Info, version 7.2.4.0 (Centers for Disease Control and Prevention, Atlanta, GA, USA) [12].

Participants were included if they had a confirmed diagnosis of MS, were aged ≥18 years, and had an EDSS score ≤ 7. The exclusion criteria were severe cognitive dysfunction or dementia, current use of an indwelling bladder catheter, pre-existing bladder conditions unrelated to MS, and refusal to provide informed consent.

### 2.2. Data Collection Tools

Two validated self-assessment questionnaires were used to assess pelvic-floor-related symptoms and their impact on QOL. The Pelvic Floor Distress Inventory (PFDI-20) is a well-established tool designed to measure the severity of symptom-related distress in individuals with PFD. It consists of three subscales: the Urinary Distress Inventory (UDI-6), which evaluates urinary symptoms; the Colorectal-Anal Distress Inventory (CRADI-8), which assesses bowel dysfunction; and the Pelvic Organ Prolapse Distress Inventory (POPDI-6), which measures symptoms associated with POP. PFDI-20 score ranges from 0 to 300, with higher scores indicating greater distress and more severe symptoms.

Similarly, the Pelvic Floor Impact Questionnaire (PFIQ-7) is a standardized tool that examines the impact of pelvic-floor-related symptoms on QOL. It includes three subscales: the Urinary Impact Questionnaire (UIQ-7), which assesses the impact of urinary symptoms; the Colorectal-Anal Impact Questionnaire (CRAIQ-7), which evaluates the impact of bowel-related symptoms; and the Pelvic Organ Prolapse Impact Questionnaire (POPIQ-7), which measures the impact of symptoms associated with POP. The total PFIQ-7 score ranges from 0 to 300, with higher scores indicating a greater impact of pelvic-floor-related symptoms on daily activities and the domains assessed by the questionnaire.

PFD was operationally assessed based on self-reported symptoms using the PFDI-20 and PFIQ-7. Participants were considered to have symptoms in a specific pelvic floor domain if they reported at least one symptom on the corresponding subscale, including the UDI-6, CRADI-8, or POPDI-6. This symptom-based operational definition was used to estimate the prevalence of pelvic-floor-related symptoms and did not represent a clinically confirmed diagnosis of PFD.

Both instruments were administered using their Arabic versions, which were linguistically adapted and evaluated for reliability among Arabic-speaking Muslim women. The Arabic PFIQ included an additional prayer-related question to reflect the cultural context of Muslim women. The questionnaires were scored according to their standardized procedures and the scoring systems established for each instrument [11,13].

### 2.3. Statistical Analysis

Data were analyzed using IBM Statistical Package for the Social Sciences SPSS, Statistics version 25 (IBM Corp., Armonk, NY, USA). Descriptive statistics were used to summarize the participants’ sociodemographic characteristics and study variables. The normality of continuous variables was assessed using the Shapiro–Wilk test.

Normally distributed continuous variables were summarized as mean ± standard deviation (SDs), whereas non-normally distributed variables were summarized as medians and interquartile ranges (IQRs). Categorical variables were summarized as frequencies and percentages. Associations between continuous variables were assessed using Spearman’s rank correlation coefficient when at least one variable was not normally distributed.

To account for multiple comparisons, *p*-values from related analyses were adjusted using the Holm–Bonferroni method. Continuous variables were retained in their original form whenever appropriate, and analyses based on arbitrary categorization of continuous variables and individual questionnaire items were removed. Therefore, the analyses focused primarily on validated overall and domain-specific questionnaire scores.

For the multivariable regression analyses, model assumptions, including linearity, multicollinearity, homoscedasticity, normality of residuals, and influential observations, were assessed using graphical and statistical diagnostics. No major violations of linearity, multicollinearity, or homoscedasticity were identified. As a degree of deviation from the normality of residuals was observed, particularly for the PFIQ-7 model, HC3 heteroscedasticity-consistent standard errors were additionally calculated in a sensitivity analysis.

## 3. Results

### 3.1. Participant Characteristics

A total of 112 Saudi women diagnosed with MS participated in this study. The mean age was 35.77 ± 8.49 years. The median time since MS diagnosis was 10 years (IQR: 12 years). The mean BMI was 25.96 ± 7.77 kg/m^2^, with body weight ranging from 37 to 170 kg. Half of the participants were married (50.0%), while 38.4% were single and 11.6% were divorced or widowed (Table 1).

### 3.2. Prevalence of PFDI-20 Symptoms

PFDI-20 scores exhibited substantial variability in symptom severity. Among the subscales, the UDI-6 had the highest mean score, followed by the CRADI-8 and POPDI-6. Overall, the total PFDI-20 scores demonstrated considerable variability in the reported burden of pelvic-floor-related symptoms (Table 2).

Overall, 104 participants reported at least one symptom within the UDI-6 subscale. The UDI-6 showed that the most commonly reported symptom was frequent urination, followed by urge incontinence, lower abdominal or genital pain, difficulty emptying the bladder, and stress UI. The least common symptom was leakage of small amounts of urine.

A total of 102 participants reported at least one symptom within the CRADI-8 subscale. Constipation was the most frequently reported symptom, followed by incomplete bowel evacuation, inability to control gas, fecal urgency, and loss of well-formed stool. The least common symptom was the loss of loose stool.

For the POPDI-6, 99 participants reported at least one symptom on the subscale. A sensation of incomplete bladder emptying was the most frequently reported symptom, followed by lower abdominal pressure or pain, vaginal heaviness, and the need for splinting to defecate. The least frequently reported symptom was splinting to void.

These findings highlight the broad spectrum of pelvic-floor-related symptoms among the participants, with urinary distress symptoms being the most prominent (Table 2).

### 3.3. Associations Between Participant Characteristics and Pelvic Floor Symptom Burden

#### 3.3.1. Correlations Between Participant Characteristics and PFDI-20 Scores

Spearman’s rank-order correlation analysis was conducted to examine the relationships between demographic characteristics and the total and subscale PFDI-20 scores. Before adjustment for multiple comparisons, age was significantly and positively correlated with all PFDI-20 subscales and the total score, indicating that older age was associated with a greater symptom burden across urinary, colorectal-anal, and prolapse-related domains. However, following the Holm–Bonferroni adjustment, only the correlations between age and UDI-6 scores and between age and the total PFDI-20 score remained statistically significant, indicating that increasing age was primarily associated with greater urinary symptom distress and a greater overall self-reported pelvic-floor-related symptom burden.

A weak positive correlation was also observed between BMI and UDI-6 scores before adjustment for multiple comparisons; however, this association did not remain statistically significant following the Holm–Bonferroni adjustment. No significant correlations were observed between BMI and the remaining PFDI-20 subscales or the total score. Similarly, the duration since MS diagnosis was not significantly correlated with any PFDI-20 subscale or the total score (Table 3).

#### 3.3.2. Multivariable Associations Between Participant Characteristics and PFDI-20 Scores

A multivariable linear regression analysis was conducted to examine the associations between demographic and clinical characteristics and total PFDI-20 scores. The model, which included age, BMI, duration since MS diagnosis, and marital status, was statistically significant overall, although it explained a relatively small proportion of the variance in PFDI-20 scores (adjusted R^2^ = 5.6%).

After adjustment for BMI, duration since MS diagnosis, and marital status, age was the only variable significantly associated with PFDI-20 scores. Each additional year of age was associated with an approximately 2.05-point increase in the total PFDI-20 score (*p* = 0.035). This association remained statistically significant when HC3 robust standard errors were applied (*p* = 0.023), indicating that the finding was robust to the use of heteroscedasticity-consistent standard errors. No statistically significant associations were observed for BMI, duration since MS diagnosis, or marital status (Table 4).

### 3.4. Impact of Pelvic-Floor-Related Symptoms on QOL

The PFIQ-7 scores varied widely, reflecting differences in the extent to which pelvic-floor-related symptoms affected participants’ QOL. Overall, 71 participants reported at least one impact within the UIQ-7 subscale, 77 participants reported at least one impact on the CRAIQ-7 subscale, and 65 participants reported at least one impact on the POPIQ-7 subscale. The UIQ-7 and CRAIQ-7 had similar mean scores, while the POPIQ-7 had a slightly lower mean score. Overall, the total PFIQ-7 scores indicated considerable variability in the impact of pelvic-floor-related symptoms on QOL (Table 5).

The UIQ-7 showed that physical activities and emotional health were the most affected domains, followed by self-esteem, household chores, and prayer. Social activities were comparatively less affected.

For the CRAIQ-7, prayer and emotional health were the domains most frequently affected by colorectal-anal symptoms, followed by self-esteem, household chores, physical activities, and social activities. Travel was comparatively less affected.

Among the POPIQ-7 domains, emotional health had the highest scores, followed by self-esteem, physical activities, and travel, whereas social activities had the lowest scores. These results suggest that emotional health and self-esteem were among the most affected areas across the subscales, emphasizing the psychological and functional burden associated with pelvic-floor-related symptoms (Table 5).

#### 3.4.1. Correlations Between Participant Characteristics and PFIQ-7 Scores

Spearman’s rank-order correlation analysis was conducted to examine the relationships between participant characteristics and the PFIQ-7 subscale and total scores. Before adjustment for multiple comparisons, age demonstrated significant positive correlations with UIQ-7 scores (ρ = 0.303, *p* = 0.001), CRAIQ-7 scores (ρ = 0.254, *p* = 0.007), POPIQ-7 scores (ρ = 0.355, *p* < 0.001), and the total PFIQ-7 score (ρ = 0.345, *p* < 0.001), indicating that older age was associated with a greater impact of pelvic-floor-related symptoms on QOL.

Following the Holm–Bonferroni adjustment for multiple comparisons, the correlations between age and UIQ-7 scores (adjusted *p* = 0.0117), POPIQ-7 scores (adjusted *p* = 0.0015), and the total PFIQ-7 score (adjusted *p* = 0.0022) remained statistically significant. However, the association between age and CRAIQ-7 scores did not remain statistically significant after adjustment. No significant correlations were observed between BMI or duration since MS diagnosis and any of the PFIQ-7 subscale or total scores (Table 6).

#### 3.4.2. Multivariable Associations Between Participant Characteristics and PFIQ-7 Scores

A multivariable linear regression analysis was conducted to examine the associations between participant characteristics and total PFIQ-7 scores. The overall model was statistically significant and explained approximately 8.8% of the variance in PFIQ-7 scores after adjustment for the number of predictors.

However, none of the individual variables included in the model were significantly associated with total PFIQ-7 scores using conventional standard errors. The comparison between single and married participants showed a relatively large negative regression coefficient (B = −32.51); however, this association did not reach statistical significance (*p* = 0.078). When HC3 robust standard errors were applied, the association remained statistically non-significant (*p* = 0.051). No statistically significant associations were observed for the remaining variables included in the model (Table 7).

### 3.5. Associations Between Participant Characteristics, Pelvic Floor Symptoms, and QOL

Age was significantly associated with several measures of pelvic-floor-related symptom burden and the impact on QOL impact. Following the Holm–Bonferroni adjustment for multiple comparisons, age remained positively correlated with UDI-6 and total PFDI-20 scores. Age also remained positively correlated with UIQ-7, POPIQ-7, and total PFIQ-7 scores after adjustment.

In the multivariable regression analysis, age was the only variable significantly associated with total PFDI-20 scores after adjustment for BMI, duration since MS diagnosis, and marital status. Each additional year of age was associated with an approximately 2.05-point increase in the total PFDI-20 score. In contrast, although the overall multivariable model for total PFIQ-7 scores was statistically significant, none of the individual predictors were significantly associated with the total PFIQ-7 score.

BMI and duration since MS diagnosis were not significantly correlated with the PFDI-20 or PFIQ-7 subscale or total scores after adjustment for multiple comparisons. Similarly, neither variable showed a statistically significant association with the respective total scores in the multivariable regression analyses. Marital status was also not significantly associated with the total PFDI-20 or PFIQ-7 scores in the multivariable models.

## 4. Discussion

Our study investigated the prevalence and impact of pelvic-floor-related symptoms among women with MS in Saudi Arabia and demonstrated a substantial burden of urinary, colorectal, anal, and prolapse-related symptoms in this population. Frequent urination (83%), urge incontinence (70.5%), and constipation (76.8%) were among the most commonly reported symptoms.

These findings are consistent with previous research linking MS-related neurological dysfunction to bladder and bowel disturbances, including impaired neural regulation and autonomic dysfunction [14].

The high frequency of urinary symptoms observed in our study is consistent with international evidence indicating that urinary symptoms affect approximately 50–90% of individuals with MS, particularly those with spinal cord involvement [15]. Similarly, colorectal-anal symptoms were frequently reported, with constipation being one of the most prevalent symptoms. This finding is consistent with the previous literature identifying neurogenic bowel dysfunction as a common manifestation of MS [15]. Collectively, these findings highlight the considerable burden of pelvic-floor-related symptoms among women with MS and underscore the importance of recognizing both urinary and bowel symptoms in this population. Comparisons between the prevalence estimates observed in the present study and those reported in previous research should be interpreted with caution. Differences in participant characteristics, recruitment methods, assessment instruments, questionnaire versions, scoring approaches, and definitions of pelvic-floor-related symptoms may substantially influence the reported prevalence estimates. Furthermore, previous studies have used different assessment methods, including self-reported questionnaires and clinical evaluations, which may limit direct comparisons across studies. Therefore, similarities or differences in prevalence estimates should be interpreted within the context of these methodological variations.

Age was the demographic characteristic most consistently associated with pelvic-floor-related symptom burden and its impact on QOL. Following adjustment for multiple comparisons, increasing age remained positively associated with urinary symptom distress and overall PFDI-20 scores. Similarly, age remained positively associated with urinary- and prolapse-related impacts on QOL, as well as with total PFIQ-7 scores.

Furthermore, in the multivariable regression analysis, age remained significantly associated with total PFDI-20 scores after adjustment for BMI, duration since MS diagnosis, and marital status. These findings suggest that older women with MS may report a greater burden of pelvic-floor-related symptoms. This association may reflect age-related changes in pelvic floor structure and function, including reductions in muscle strength and connective tissue support, as well as the potential contribution of neurological impairment associated with MS [16]. However, given the cross-sectional design of the study, these findings should be interpreted as associations and should not be considered evidence of a temporal or causal relationship.

Although increasing age was statistically associated with higher total PFDI-20 scores, the magnitude of this association was modest, with each additional year of age corresponding to an approximately 2.05-point increase in the total score. This effect should be interpreted with caution in relation to the reported minimal clinically important difference (MCID) of 45 points for the PFDI-20, as this threshold was developed primarily to evaluate meaningful changes in patient-reported outcomes and may not directly apply to cross-sectional differences between individuals [17]. Similarly, the multivariable models explained only a modest proportion of the variance in PFDI-20 and PFIQ-7 scores, highlighting that statistical associations may not necessarily reflect clinically meaningful differences. The reported MCID for the PFIQ-7 is 36 points (12%); however, the applicability of this threshold to cross-sectional associations remains uncertain [17]. Therefore, the clinical relevance of the observed associations should be interpreted with caution.

While an association between marital status and urinary symptom severity was observed in the initial unadjusted analysis, this relationship was not retained in the multivariable analysis. Therefore, marital status cannot be interpreted as a factor independently associated with urinary symptom burden in the present study. The initially observed association may have been influenced by unmeasured reproductive and hormonal factors, including parity, history of pregnancy and childbirth history, mode of delivery, and hormonal status, which are recognized contributors to pelvic floor symptoms [18]. As these variables were not assessed, their potential confounding effects could not be evaluated. Accordingly, the relationship between marital status and urinary symptoms should be interpreted with caution, and further studies incorporating relevant reproductive and clinical characteristics are needed to clarify this relationship.

A weak positive association between BMI and UDI-6 was observed in the initial unadjusted correlation analysis; however, this association did not remain statistically significant after adjustment for multiple comparisons. BMI was also not significantly associated with overall PFDI-20 or PFIQ-7 scores in the multivariable analyses. These findings contrast with previous reports suggesting that higher BMI may contribute to pelvic-floor-related symptoms through increased intra-abdominal pressure. However, pelvic-floor-related symptoms are multifactorial, and factors beyond BMI, including pelvic floor muscle function, physical activity, reproductive history, and hormonal status, may contribute to the overall symptom burden [11].

Similarly, duration since MS diagnosis was not significantly associated with PFDI-20 or PFIQ-7 scores. Although previous studies have suggested that longer disease duration may be associated with progressive neurological complications [19], our results indicate that time since diagnosis alone may not adequately capture the disease-related factors associated with pelvic-floor-related symptom burden. Other characteristics, such as lesion location, MS phenotype, and severity of neurological disability, may be more relevant but were not examined in the present study.

The PFIQ-7 findings further demonstrated the impact of pelvic-floor-related symptoms on participants’ QOL. Limitations in physical activities and emotional well-being were among the most frequently reported areas of impact, indicating that these symptoms may extend beyond physical discomfort and affect daily functioning, physical activity, and the psychosocial aspects of participants’ lives. These findings are consistent with previous research highlighting the substantial physical, emotional, and social burden associated with pelvic-floor-related symptoms, particularly among women with neurological disorders [19].

### 4.1. Clinical Implications and Recommendations

Our findings support the consideration of routine assessment of pelvic-floor-related symptoms among women with MS, particularly given the substantial burden of urinary and colorectal-anal symptoms observed in this population. Increasing age was associated with greater self-reported pelvic-floor-related symptom burden in the present sample, suggesting that age may be an important characteristic to consider when evaluating these symptoms.

Pelvic floor rehabilitation may be considered as a first-line conservative intervention and may include pelvic floor muscle training, bladder retraining, bowel management programs, biofeedback, patient education, and multidisciplinary neurorehabilitation. Evidence from previous studies suggests that pelvic floor muscle training can improve pelvic floor muscle strength, endurance, and coordination, leading to reductions in urinary symptoms and improvements in QOL [20]. Additional physiotherapy interventions, including electromyographic biofeedback, intravaginal neuromuscular electrical stimulation, and transcutaneous tibial nerve stimulation, have demonstrated beneficial effects on lower urinary tract symptoms, continence outcomes, and patient-reported QOL in individuals with MS [21].

Furthermore, lifestyle modifications, including adequate hydration, optimization of dietary fiber intake, and regular bowel routines, may help alleviate colorectal-anal symptoms. Given the symptom burden observed in this study, multidisciplinary assessment and management involving neurologists, physiotherapists, urologists, and continence specialists may be appropriate when clinically indicated.

In addition, culturally tailored education programs may enhance awareness, reduce stigma, promote early intervention, and facilitate engagement with appropriate assessment and rehabilitation services [22].

### 4.2. Limitations and Future Directions

This study has several limitations. First, the cross-sectional design precludes causal or temporal inferences from the observed associations, and the use of self-reported data may have introduced recall and reporting bias.

Additionally, participants were recruited through MS societies, which may have resulted in selection bias. Individuals actively engaged with patient organizations may differ from the broader population of women with MS in terms of symptom awareness, healthcare utilization, and willingness to report pelvic-floor-related symptoms.

Furthermore, several potentially important confounding factors were not collected or considered, including parity and obstetric history, menopausal and hormonal status, prior pelvic surgery, comorbidities, MS phenotype, detailed measures of neurological disability, physical activity, and medication use. Although age, BMI, duration since MS diagnosis, and marital status were included in the multivariable analyses, residual confounding cannot be excluded. Future studies should incorporate these clinically relevant factors and use longitudinal designs to further examine the determinants and progression of pelvic-floor-related symptoms among women with MS.

Recruitment through MS societies facilitated access to women with MS across different regions of Saudi Arabia; however, this sampling approach may have introduced selection bias and non-response bias. Participation was voluntary, and women affiliated with MS societies or those who chose to respond to the online questionnaire may differ from those who were not contacted or did not participate. Therefore, the findings may not be fully representative of all Saudi women with MS, and their generalizability should be interpreted with caution.

## 5. Conclusions

This study demonstrates a substantial burden of self-reported pelvic-floor-related symptoms among women with MS in Saudi Arabia. Frequent urination (83%), urge incontinence (70.5%), and constipation (76.8%) were among the most commonly reported symptoms. These symptoms were also associated with a considerable impact on QOL, particularly with respect to physical activity and emotional well-being.

Increasing age was the factor most consistently associated with greater pelvic-floor-related symptom burden. After adjustment for multiple comparisons, age remained associated with greater urinary symptom distress, higher overall PFDI-20 scores, and greater urinary- and prolapse-related impact on QOL. In the multivariable analysis, age remained significantly associated with total PFDI-20 scores after adjustment for BMI, duration since MS diagnosis, and marital status.

In contrast, BMI and duration since MS diagnosis were not significantly associated with PFDI-20 or PFIQ-7 scores in the adjusted analyses. Marital status was also not significantly associated with total PFDI-20 or PFIQ-7 scores in the multivariable models. Overall, these findings highlight the importance of recognizing and assessing pelvic-floor-related symptoms among women with MS and support the need for further research incorporating relevant reproductive, hormonal, and disease-related characteristics to better understand the factors associated with symptom burden in this population.

## Figures and Tables

**Table 1:** Participant demographics.

	Mean (±Std. Deviation) or Median (IQR)	Minimum	Maximum
Age (years)	35.77 (±8.491)	18	55
Duration Since MS Diagnosis (years)	10 (12)	0	46
Weight (Kg)	65.00 (±16.5)	37.00	170.00
Height (Cm)	159.00 (8)	132.00	176.00
BMI (Kg/m^2^)	25.96 (7.77)	17.21	69.88
		Count	Column N%
Marital Status	Single	43	38.4%
	Married	56	50.0%
	Divorced/Widowed	13	11.6%
Mean (±Std. Deviation) Median (Interquartile Range)			

**Table 2:** Descriptive statistics of PFDI-20 symptoms.

	Mean (±Std. Deviation)	Prevalence	95% CI	Rank
**UDI-6**	38.00 (±25.98)	104 (92.9)	[86.5–96.3]	
Frequency		93 (83.0)	[76.1–90.0]	1
Urge incontinence		79 (70.5)	[61.5–78.2]	2
Pain		72 (64.3)	[55.1–72.6]	3
Difficulty		71 (63.4)	[54.2–71.7]	4
Stress urinary incontinence		67 (59.8)	[50.6–68.4]	5
Drops		45 (40.2)	[31.6–49.4]	6
**CRADI-8**	29.87 (±24.36)	102 (91.1)	[84.3–95.1]	
Constipation		86 (76.8)	[68.2–83.6]	1
Sense of incomplete bowel evacuation		77 (68.8)	[59.7–76.7]	2
Lose gases		67 (59.8)	[50.6–68.4]	3
Fecal urgency		61 (54.5)	[45.2–63.4]	4
Lose well-formed stools		59 (52.7)	[43.5–61.7]	5
Painful defecation		54 (48.2)	[39.2–57.4]	6
Rectal prolapse		42 (37.5)	[29.1–46.7]	7
Lose non-well-formed stools		34 (30.4)	[22.6–39.4]	8
**POPDI-6**	28.77 (±24.06)	99 (88.4)	[81.1–93.1]	
Sense of incomplete bladder evacuation		85 (75.9)	[67.2–82.9]	1
Pressure, pain in the lower abdomen		72 (64.3)	[55.1–72.6]	2
Heaviness, vaginal		65 (58.0)	[48.8–66.8]	3
Splint to defecate		55 (49.1)	[40.0–58.2]	4
Something protruding from vaginal		34 (30.4)	[22.6–39.4]	5
Splint to void		20 (17.9)	[11.9–26.0]	6
**PFDI-20**	96.64 (±66.57)			
**Frequency (prevalence)** **95% CI [Lower bound–Upper bound]**				

**Table 3:** PFDI-20 correlations.

Predictor	Outcome Measures	Spearman ρ	Unadjusted *p*-Value	pHolm
Age	UDI-6	0.334	<0.001	0.0039
	CRADI-8	0.216	=0.022	0.219
	POPDI-6	0.209	=0.027	0.244
	PFDI-20 Total Score	0.298	=0.001	0.0155
BMI	UDI-6	0.194	=0.041	0.325
	CRADI-8	−0.046	=0.628	1.000
	POPDI-6	−0.014	=0.881	1.000
	PFDI-20 Total Score	0.066	=0.491	1.000
MS diagnosis duration	UDI-6	0.111	=0.243	1.000
	CRADI-8	0.089	=0.353	1.000
	POPDI-6	−0.029	=0.761	1.000
	PFDI-20 Total Score	0.084	=0.381	1.000

**Table 4:** Multiple linear regression for PFDI-20.

Predictor	B	Conventional SE	Conventional *p*	HC3 SE	HC3 *p*
Intercept	13.584	40.077	0.735	35.715	0.704
Age	2.052	0.958	0.0345	0.892	0.0233
BMI	0.663	0.806	0.413	0.628	0.294
MS diagnosis duration	−0.292	0.620	0.638	0.598	0.626
Single **vs.** Married	−9.989	15.844	0.530	13.446	0.459
Divorced/Widowed **vs.** Married	−7.722	20.526	0.708	24.348	0.752

Model statistics: R^2^ = 0.099; adjusted R^2^ = 0.056; F(5,106) = 2.317, *p* = 0.049.

**Table 5:** Impact of Pelvic-floor-related symptoms on QOL.

	Mean (±Std. Deviation)	Prevalence	95% CI	Rank
**UIQ-7**	24.49 (±29.14)	71 (63.4)	[54.2–71.7]	
Physical activities		54 (48.2)	[39.2–57.4]	1.5
Emotional health		54 (48.2)	[39.2–57.4]	1.5
Low self-esteem		52 (46.4)	[37.5–55.6]	3
Household chores		47 (42.0)	[33.2–51.2]	4
Prayer		43 (38.4)	[29.9–47.6]	5
Travel		41 (36.6)	[28.3–45.8]	6
Social activities		39 (34.8)	[26.6–44.0]	7
**CRAIQ-7**	25.38 (±27.44)	77 (68.7)	[60.2–77.3]	
Prayer		63 (56.3)	[47.0–65.1]	1.5
Emotional health		63 (56.3)	[47.0–65.1]	1.5
Low self-esteem		56 (50.0)	[40.9–59.1]	3
Household chores		46 (41.1)	[32.4–50.3]	4.5
Physical activities		46 (41.1)	[32.4–50.3]	4.5
Social activities		44 (39.3)	[30.2–49.0]	6
Travel		41 (36.6)	[28.3–45.8]	7
**POPIQ-7**	20.59 (±27.37)	65 (58.0)	[48.8–66.8]	
Emotional health		54 (48.2)	[39.2–57.4]	1
Low self-esteem		47 (42.0)	[33.2–51.2]	2
Physical activities		43 (38.4)	[29.9–47.6]	3
Travel		35 (31.3)	[23.4–40.3]	4
Household chores		33 (29.5)	[21.8–38.5]	5
Social activities		32 (28.6)	[21.0–37.5]	6
**PFIQ-7**	70.46 (±78.01)			
Frequency (prevalence) 95% CI [Lower bound–Upper bound]				

**Table 6:** PFIQ-7 Correlation.

Predictor	Outcome Measures	Spearman ρ	Unadjusted *p*-Value	pHolm
Age	UIQ-7	0.303	=0.001	0.0117
	CRAIQ-7	0.254	=0.007	0.0618
	POPIQ-7	0.355	<0.001	0.0015
	PFIQ-7 Total Score	0.345	<0.001	0.0022
BMI	UIQ-7	0.122	=0.201	1.000
	CRAIQ-7	−0.024	=0.805	1.000
	POPIQ-7	−0.006	=0.946	1.000
	PFIQ-7 Total Score	0.027	=0.781	1.000
MS diagnosis duration	UIQ-7	0.071	=0.456	1.000
	CRAIQ-7	0.117	=0.219	1.000
	POPIQ-7	0.115	=0.228	1.000
	PFIQ-7 Total Score	0.130	=0.172	1.000

**Table 7:** Multiple linear regression for PFIQ-7.

Predictor	B	Conventional SE	Conventional *p*	HC3 SE	HC3 *p*
Intercept	5.983	46.153	0.897	41.407	0.885
Age	1.280	1.104	0.249	0.911	0.163
BMI	0.826	0.929	0.376	1.169	0.481
MS diagnosis duration	0.681	0.714	0.342	0.900	0.451
Single **vs.** Married	−32.510	18.246	0.078	16.441	0.051
Divorced/Widowed **vs.** Married	3.282	23.637	0.890	26.470	0.902

**Model statistics:** R^2^ = 0.130; adjusted R^2^ = 0.088; F(5,106) = 3.153, *p* = 0.011.

## Data Availability

Data will be available upon request. The data are not publicly available because they contain information that could compromise the privacy and confidentiality of the study participants and are subject to institutional ethical restrictions.

## Abbreviations

The following abbreviations are used in this manuscript:

MS	Multiple sclerosis
EDSS	Expanded Disability Status Scale
PFD	Pelvic Floor Dysfunction
QOL	Quality Of Life
PFDI	Pelvic Floor Distress Inventory
PFIQ	Pelvic Floor Impact Questionnaire
CNS	Central Nervous System
UI	Urinary Incontinence
OAB	Overactive Bladder
FI	Fecal Incontinence
POP	Pelvic Organ Prolapse
UIQ	Urinary Impact Questionnaire
CRAIQ	Colorectal-Anal Impact Questionnaire
POPIQ	Pelvic Organ Prolapse Impact Questionnaire

## References

[1] Karussis D. (2014). The Diagnosis of Multiple Sclerosis and the Various Related Demyelinating Syndromes: A Critical Review. J. Autoimmun..

[2] Walton C., King R., Rechtman L., Kaye W., Leray E., Marrie R.A., Robertson N., La Rocca N., Uitdehaag B., van der Mei I. (2020). Rising Prevalence of Multiple Sclerosis Worldwide: Insights from the Atlas of MS, Third Edition. Mult. Scler. J..

[3] Fang X., Patel C., Gudesblatt M. (2020). Multiple Sclerosis: Clinical Updates in Women’s Health Care Primary and Preventive Care Review. Obstet. Gynecol..

[4] AlJumah M., Bunyan R., Al Otaibi H., Al Towaijri G., Karim A., Al Malik Y., Kalakatawi M., Alrajeh S., Al Mejally M., Algahtani H. (2020). Rising Prevalence of Multiple Sclerosis in Saudi Arabia, a Descriptive Study. BMC Neurol..

[5] Dobson R., Giovannoni G. (2019). Multiple Sclerosis—A Review. Eur. J. Neurol..

[6] Macías Islas M.Á., Ciampi E. (2019). Assessment and Impact of Cognitive Impairment in Multiple Sclerosis: An Overview. Biomedicines.

[7] Demir S. (2022). Expanded Disability Status Scale (EDSS) in Multiple Sclerosis. Cam Sakura Med. J..

[8] Sen S. (2018). NEUROSTATUS AND EDSS CALCULATION WITH CASES. Arch. Neuropsychiatry.

[9] Sarıibrahim Astepe B., Köleli I. (2019). Translation, Cultural Adaptation, and Validation of Australian Pelvic Floor Questionnaire in a Turkish Population. Eur. J. Obstet. Gynecol. Reprod. Biol..

[10] Kenne K.A., Wendt L., Brooks Jackson J. (2022). Prevalence of Pelvic Floor Disorders in Adult Women Being Seen in a Primary Care Setting and Associated Risk Factors. Sci. Rep..

[11] Al-Badr A., Saleem Z., Kaddour O., Almosaieed B., Dawood A., Al-Tannir M., AlTurki F., Alharbi R., Alsanea N. (2022). Prevalence of Pelvic Floor Dysfunction: A Saudi National Survey. BMC Women’s Health.

[12] Fleiss J.L., Levin B., Paik M.C. (2013). Statistical Methods for Rates and Proportions.

[13] El-Azab A.S., Abd-Elsayed A.A., Imam H.M.K. (2009). Patient Reported and Anatomical Outcomes after Surgery for Pelvic Organ Prolapse. Neurourol. Urodyn..

[14] Pintér A., Cseh D., Sárközi A., Illigens B., Siepmann T. (2015). Autonomic Dysregulation in Multiple Sclerosis. Int. J. Mol. Sci..

[15] Magnuson F., Christensen P., Krassioukov A., Rodriguez G., Emmanuel A., Kirshblum S., Krogh K. (2023). Neurogenic Bowel Dysfunction in Patients with Spinal Cord Injury and Multiple Sclerosis—An Updated and Simplified Treatment Algorithm. J. Clin. Med..

[16] Sparaco M., Bonavita S. (2022). Pelvic Floor Dysfunctions and Their Rehabilitation in Multiple Sclerosis. J. Clin. Med..

[17] Barber M.D., Walters M.D., Bump R.C. (2005). Short Forms of Two Condition-Specific Quality-of-Life Questionnaires for Women with Pelvic Floor Disorders (PFDI-20 and PFIQ-7). Am. J. Obstet. Gynecol..

[18] Afshari P., Abedi P., Majdinasab N., Tafakh S., Haghighizadeh M. (2025). Strengths of Pelvic Floor Muscles in Women with Multiple Sclerosis and Its Relationship with Urinary Incontinence and Quality of Life. Front. Neurol..

[19] Lin S.D., Butler J.E., Boswell-Ruys C.L., Hoang P., Jarvis T., Gandevia S.C., McCaughey E.J. (2019). The Frequency of Bowel and Bladder Problems in Multiple Sclerosis and Its Relation to Fatigue: A Single Centre Experience. PLoS ONE.

[20] Curillo-Aguirre C.A., Gea-Izquierdo E. (2023). Effectiveness of Pelvic Floor Muscle Training on Quality of Life in Women with Urinary Incontinence: A Systematic Review and Meta-Analysis. Medicina.

[21] Lúcio A., D’ancona C.A.L., Perissinotto M.C., McLean L., Damasceno B.P., De Moraes Lopes M.H.B. (2016). Pelvic Floor Muscle Training With and Without Electrical Stimulation in the Treatment of Lower Urinary Tract Symptoms in Women with Multiple Sclerosis. J. Wound Ostomy Cont. Nurs..

[22] Hay-Smith E.J.C., Starzec-Proserpio M., Moller B., Aldabe D., Cacciari L., Pitangui A.C.R., Vesentini G., Woodley S.J., Dumoulin C., Frawley H.C. (2024). Comparisons of Approaches to Pelvic Floor Muscle Training for Urinary Incontinence in Women. Cochrane Database Syst. Rev..

